# Analysis of Hygrothermal Microclimatic (HTM) Parameters in Specific Food Storage Environments in Slovakia

**DOI:** 10.3390/ijerph17062092

**Published:** 2020-03-21

**Authors:** Iveta Marková, Ivana Tureková, Jana Jaďuďová, Emília Hroncová

**Affiliations:** 1 Department of Fire Engineering, Faculty of Security Engineering, University of Žilina, Univerzitná 1, 010 26 Žilina, Slovakia; 2 Constantine the Philosopher University in Nitra, Tr. A. Hlinku 1, 949 74 Nitra, Slovakia; iturekova@ukf.sk; 3 Matej Bel University, Faculty of Natural Sciences, Department of Environmental Management, Tajovskeho 40, 97401 Banska Bystrica, Slovakia; jana.jadudova@umb.sk; 4 European Science and Research Institute, A. Hlinku 29, 96001 Zvolen, Slovakia

**Keywords:** hygrothermal microclimatic (HTM) parameters, food storage environment

## Abstract

The quality of work environment, temperature changes and humidity must be controlled in every production process and in the locations where employees are present. The aim of this paper is to objectively assess the exposure of employees to microclimatic factors of the workplace environment: the warehouse, changing rooms, office and cold room refrigerator. Data were obtained in real working conditions. The heat stress due to cold and heat exposure in the individual locations was evaluated using the WBGT (wet bulb globe temperature) indicator. The parameters of the hygrothermal microclimate (HTM) were objectified by a QUES Temp 44/46 T spherical thermometer. The measurements were performed both in cold and hot periods of the year. The measurements confirmed standard temperatures for individual types of interiors in the winter period, but in the summer period there was a variability of results, leading to the thermal discomfort of employees. The assessment of the WBGT index revealed that nearly 80% of employees are susceptible to hypothermia as a result of thermal stress conditions. It was proven that the temperatures measured by a spherical thermometer in the hottest room were 8.62% higher than the calculated operating temperature, while the difference in the cold room refrigerator was only 1.28% higher.

## 1. Introduction

The indoor environment of buildings where an employee works represents the living space of each employee. Adherence to the quality of indoor environmental parameters has come to the forefront in the recent period, when the climate change is most obviously manifested by an extreme increase in air temperature [1,2,3].

The hygrothermal microclimate is determined by temperature, humidity and air velocity, and by stereo temperature, it also significantly affects working conditions [4,5]. The parameters of the indoor environment of buildings can be optimized by regulating the hygrothermal parameters.

Assessing the hygrothermal microclimate HTM in the work environment is difficult because [6]:

- Although individual factors can be qualified and quantified separately, HTM is a complex of variables in interaction with each other.
- Assessment of the HTM is a function of exposure time and type of work activity.
- There are different individual reactions of people to the qualitative and quantitative effects of HTM factors.
- The effects of HTM can be somewhat “mitigated” by human adaptation or acclimatization.

The assessment of the risk of human exposure to heat and cold is a very important topic of the present. Additionally, in the context of global warming, such risk assessment for occupational exposure and for the general public health has gained interest. Over the last 70+ years, a range of over 160 different climatic stress indices have been developed [7] of which over 100 are for heat stress. Of all the empirical or direct indices reviewed by Goldman [8], only the WBGT, the wet bulb globe temperature index, is still in widespread use for heat stress management, as shows the study by Havenith and Fiala [9].

The research of an index for the assessment of heat stress in workplaces is still a much debated topic, as confirmed by the impressive number of studies and indices that have appeared in the literature of the last 50 years [10,11,12,13]. d’Ambrosio et al. [14] made of conclusion that nowadays, two methods apparently are approved internationally: the WBGT index [15] and the predicted heat strain (PHS) model [14,16]. The chosen method in this work was the method: index WBGT, with respect to the methodology used in Slovakia.

Thermal comfort is defined as the condition of mind that expresses satisfaction with the thermal environment [17]. Two kinds of approach exist in contemporary thermal comfort research: they are heat balance models based on laboratory studies and adaptive models based on field studies [18,19].

Thermal comfort standards determine the energy consumption by a building’s environmental systems, therefore they play an important role in building sustainability. International standards such as ISO 7730 [20] and the ASHRAE Standard 55–92 [17] define comfort zones, by applying Fanger’s lab-based method, which was described and applied in the PMV–PPD method [21]. The PMV-PPD model is a widely used design tool incorporated in thermal comfort standards [17,20], that suggests it applies equally to different building types (schools, commercial, hospitals, etc.) and climate [21,22,23,24,25]. The predicted mean vote (PMV) model stands among the most recognized thermal comfort models. It was developed using the principles of heat balance and experimental data collected in a controlled climate chamber under steady state conditions. The PMV-PPD model is applied globally but does not directly take into account the adaptation mechanisms and outdoor thermal conditions [26,27,28].

Thermal comfort is determined by factors affecting heat exchange between the human body and its surroundings. They fall into two basic categories, namely environmental and personal factors [29,30,31,32,33].

Environmental factors are; air temperature *t_a_* (°C), which is the indoor air temperature without the effect of surrounding radiant heat sources, air velocity *v_a_* (m·s^–1^) is a factor determined by its size and direction, mean radiant temperature *t_r_* (°C) is the uniform temperature of an imaginary enclosure in which the radiant heat transfer from the human body is equal to the radiant heat transfer in the actual non-uniform enclosure and the relative humidity *RH*, the effect of which is relatively low at a relative humidity of air between 30 to 70% [34].

Personal factors are human energy expenditure *M* (W·m^–2^) or met (1 met = 58.2 W·m^–2^), which indicates a person’s heat output dependent on their physical activity and personal disposition (age, figure, physical condition), and the conditions in which the person is located, thermal resistance of the garment affecting heat transfer from the human body to the surroundings; for the purpose of thermal comfort studies, a unit named clo (1 clo = insulation mass with thermal resistance *R_c_*_l_ = 0.155 m^2^·K·W^–1^) was introduced [35,36,37].

Human thermoregulatory conditions are met by ensuring appropriate workplace temperatures. The optimal and acceptable workplace temperature in the Slovak Republic is set based on the type of performed work as written in the Work classification derived from Decree 99/2016 Coll. and Decree 259/2008 Coll. [38,39].

Alternatively, the thermal stress of employees can be evaluated using the WBGT (wet bulb globe temperature) method. Based on the WBGT index [40], the American Conference of Government Industrial Hygienists (ACGIH) published the “permissible heat exposure threshold limits values (TVL)”, which refer to those heat stress conditions under which nearly all workers may be repeatedly exposed without adverse health effects [41].

The WBGT method is designed to evaluate the average effect of heat on human bodies during their activity [42,43]. It is not suitable for the evaluation of heat stress over very short periods of time with significant changes in conditions, for the evaluation of optimal and acceptable microclimate conditions, or for the evaluation of directional effects of thermal radiation sources. The index is useful for assessing extreme temperatures in the work environment [44,45]. Its resulting value depends on three parameters: the globe thermometer, spherical thermometer and the air temperature. The WBGT thermal stress indicator is determined by measuring the temperature of a wet and spherical thermometer in indoor and outdoor areas without sunlight, according to [46]:

(1) $$WBGT=0.7\;t_{w}+0.3\;t_{g}$$

where *t_g_* is the output value measured by a spherical thermometer in °C (according [15]), which includes the effect of the simultaneous influence of air temperature, ambient temperature and air velocity; *t_w_* is the temperature of the naturally ventilated wet thermometer in °C without forced ventilation.

An inherent limitation of the WBGT is its applicability across a broad range of potential scenarios and environments because of measuring *t_g_*. The black-globe temperature is measured by a temperature sensor placed in the center of a thin copper matt-black globe (diameter: 150 mm). In many circumstances, measuring *t_g_* is cumbersome and impractical [47,48,49]. The WBGT is still the index adopted by the international authorities [50,51,52].

The aim of this paper was to objectify and evaluate the HTM factors affecting work in food storage environment in winter and more importantly in summer, when the employees are exposed to cold. The operating temperature *t_o_*, which is dependent on the measured parameters of air temperature *t_a_*, relative air humidity and air velocity, was used as an assessment criterion of the effect of the thermal environment on the human body, i.e., for the assessment of the thermal stress on the human body.

The operating temperature is the temperature of the enclosed uniform isothermal black area (in the black location), in which the same amount of heat exchange by convection or radiation would occur between the human body and the environment, as in a real non-homogeneous environment. The range of optimal and acceptable values of the operating temperature and other microclimatic variables depends on the total energy expenditure of the organism (work classes), and is determined for the warm and cold period of the year. The difference between warm and cold periods is related to the differences in clothing (clothing insulation in clo; in summer 0.3 to 0.7 clo and in winter 1.0 to 1.5 clo) [53].

The result is an assessment of whether HTM factors in the non-standard food storage environment meet the limits for acceptable and optimal conditions for a given type of work. In practice, however, it is not enough to only define compliance/non-compliance with the working conditions, but also to define requirements to ensure that the required limit values are met, which was also the application output of the measured values.

## 2. Materials and Methods 

**Figure 1:**
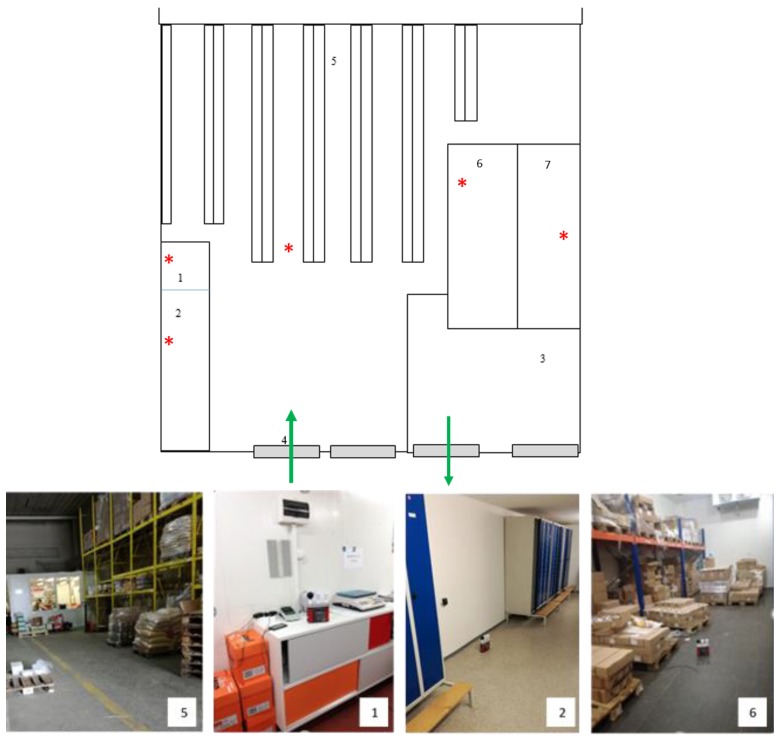
Ground plan of the warehouse with experimental points. Legend: 1—Office in the warehouse; 2—Changing rooms and toilets inside the warehouse; 3—Expedition place; 4—Loading ramp; 5—Storage room; 6—Cold room refrigerator in the warehouse, 7—Freezer room; *—experimental point.

The storage of food products, including premises accessible to employees which are related to their work activities, was subject to the objectification of the hygrothermal microclimate by the [42] (Table 1 and Figure 1). 

The air exchange in the warehouse is combined—natural replacement is ensured by open cargo gates, forced is ensured by artificial fans NV 400, with air flow of 25 m^3^·min^−1^, located on the ceiling of the warehouse. The toilets have their own local exhaust system. The other objects have natural ventilation.

Employees work in a one-shift operation in an eight-hour shift (06:00–14:30), with a thirty-minute break after four hours of continuous work. These are acclimatized employees performing long-term work. Work activities in the office are administrative in nature; on the other hand, 80% of the employees work in the warehouse, where the activity of employees is related to logistics, mainly the loading and unloading of goods using forklifts.

The measurement of internal factors required the objectivization of environmental factors around the assessed buildings [54]. 

**Figure 2:**
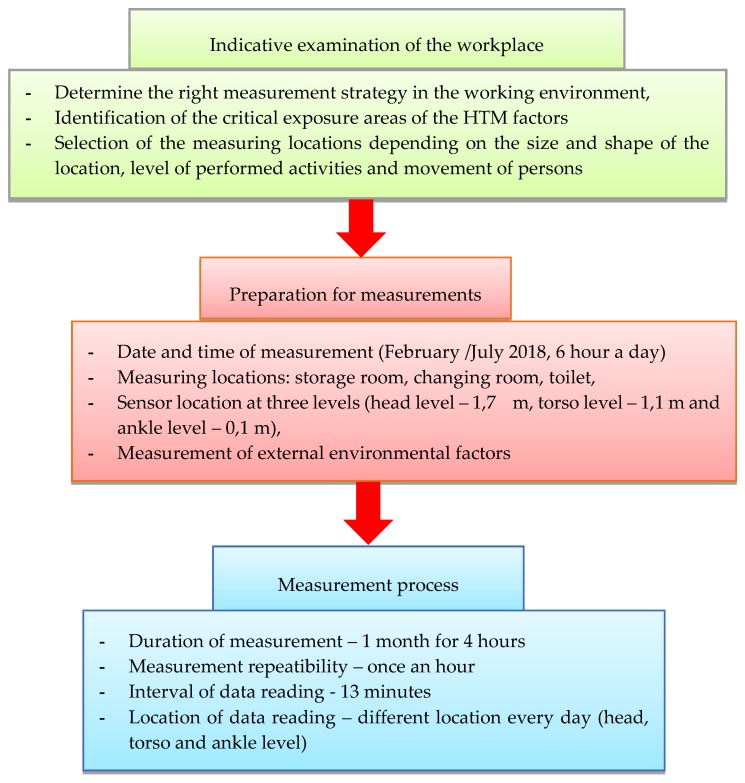
Planned experiment process.

The measurement and evaluation of HTM was performed in accordance with [38,55,56] and ISO 7730 [20], and according to Frič et al. [56] The preparation and procedure of the HTM measurement was carried out in accordance with the following scheme (Figure 2). Measurements are made in the hot and cold season of the year. By definition [55] and Kraliková and Sokolová [57], the hot period of the year is a period with an average daily outdoor temperature of 13 °C and above. If the average daily temperature drops below 13 °C for two consecutive days, it is considered the cold season of the year. The coldest month was February, the hottest July, when measurements were also taken. These measurements were conducted on the basis of employees’ suggestions, who felt thermal discomfort mainly in the environment of the cold store. The second reason was the restrictive measures in the legislation that came into force in 2019.

The values measured within the experiments or calculated according to the relationships (2)–(4) were assessed and compared. The results were evaluated under optimal and acceptable microclimate conditions. In terms of vertical distribution of the microclimatic parameters, the measurement was performed in a heterogeneous environment (with variable air velocity, with cooler flooring in hot environment) and the resulting parameters: globe thermometer temperature, mean radiant temperature and air velocity were measured at all three height levels and they were calculated as the average of the measured values, according to Formula (2):

(2) $$\emptyset x=\frac{\chi_{head}+\chi_{torso}+\chi_{ankles}}{3}$$

Frič et al. [56] explain these calculations according to the applicable OOFŽP/268/2013 methodological procedure [55] in the following steps:

The mean radiant temperature *t_r,m_*, which is needed to calculate the operating temperature, is expressed as (3):

(3) $$t_{r,m}={\left|{\left(t_{g}+273\right)}^{4}+2.5\cdot 10^{8}\;\upsilon_{a}^{0.6}\;\left(t_{g}-t_{a}\right)\;\right|}^{0.25}-273$$

where *t_g_* is the resulting globe thermometer temperature (GLOBE) in °C, *t_a_* is the air temperature (DRY) in °C, and υ_a_ is the air velocity in m·s^−1^.

The value of the operating temperature t_o_ is calculated from directly measurable physical quantities according to Formula (4):

(4) $$t_{0}=\frac{t_{a\;}{\left(10\cdot \upsilon_{a}\right)}^{\frac{1}{2}}+t_{r,m}}{1+{\left(10\cdot \upsilon_{a}\right)}^{\frac{1}{2}}}$$

**Figure 3:**
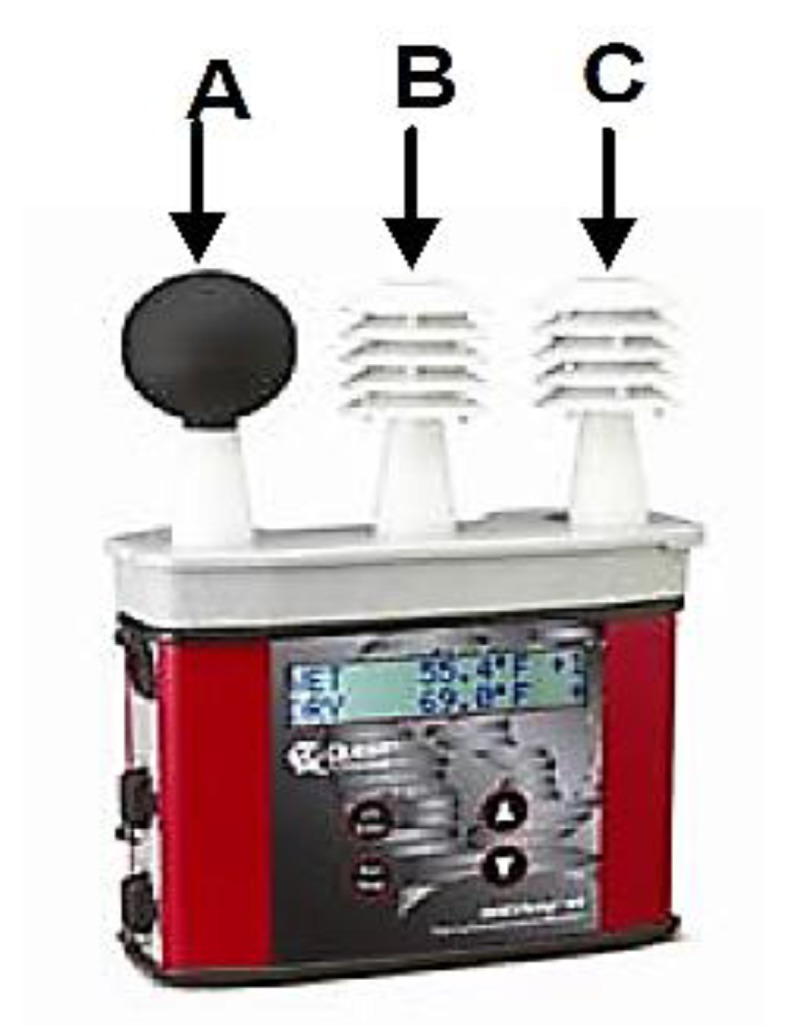
QUEST Temp 44/46 TM gauge-sensors identified: A is Globe thermomether, B is Relative humidity sensor and C is Dry bulb thermomether. Note: Mentioning the accuracy of the sensors used: Sensor Types: Temperature: 1000 ohm platinum Resistance Temperature Detector RTD, Humidity: Integrated circuit with capacitive polymer sensor Accuracy, Dry Bulb and Globe Temperature: +/−0.5 °C between 0°C and 120 °C, Waterless Wet Bulb Temperature: Expanded measurement uncertainty of 1.1 °C (k = 2) between 0 °C and 80 °C, Relative humidity: +/−5% between 20 to 95% (non-condensing), Operating Temperature Range Sensor Assembly: −5 °C to +100 °C Electronics: −5 °C to 60 °C.

The 3 MTM QUES Temp 44/46 TM (Figure 3) was used to measure the microclimatic parameters in selected indoor locations, to monitor the thermal stress out in accordance with the following scheme (Figure 3): 

## 3. Results

Microclimatic conditions were measured according to the prepared time schedule. The first measurements were carried out in February 2018, with the average outside atmospheric temperature being 4.2 °C during the respective week. In the office, the temperature of 20 °C was maintained by a local electrical heat source (heater). The temperature in the changing rooms and toilets inside the warehouse was 15 °C, the warehouse temperature was 15.5 °C ± 0.5 °C. The temperature in the cold room refrigerator was 5.5 °C ± 0.5 °C, which was in accordance with the recommended values of 0–8 °C.

**Figure 4:**
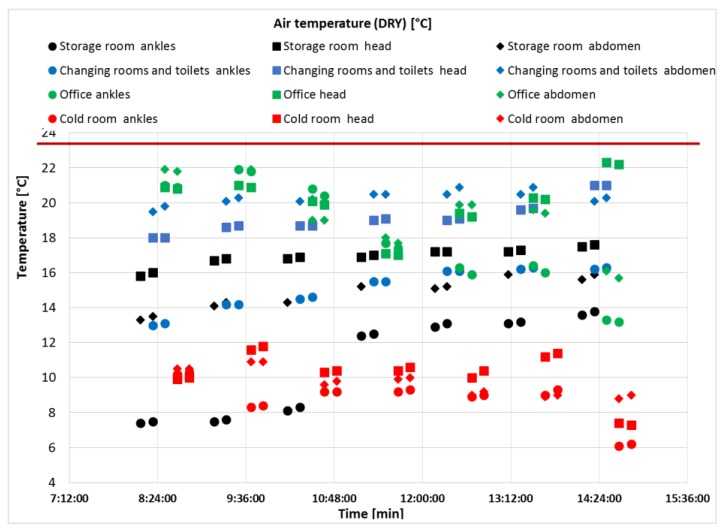
Air temperature measured at different height levels in the objectified locations. Legends: red horizontal line represents the average outside atmospheric temperature (ambient temperature).

In June 2018, the average atmospheric temperature over the respective week was 23.6 ± 2.5 °C. The average outside atmospheric temperature is indicated by a red line in Figure 4 (data are obtained from monitoring of mobile weather application and data are provided by Slovak Hydrometeorological Institute in Bratislava, Slovakia). For that reason, the variability of the measured results is small (Figure 3), as the storage area is ventilated through permanently open industrial doors. They were installed in a straight line with the building wall, therefore there is no draught inside the warehouse and the air flow rate was 0.2 m s^−1^.

In terms of health risks, the employees fall under work category 2a. It is a long-term performance of work activities in the indoor workplace, where the ambient temperature does not drop below 4 °C [58]. Figure 4 shows the course of the average dry temperature inside the organization. Figure 4 presents the average daily temperatures measured at the same time during the month of July on 20 working days. 

The course of the measured temperatures at the individual height levels (1.7 m; 1.1 m; 0.1 m) was relatively consistent, in line with Králiková et al. [59] Differences in temperatures at different height levels were only noted in the changing rooms and storage room. The temperature at the torso level was higher than at the head level in changing rooms and toilets after last work’s hour. 

The influence of external weather conditions was reflected in changes in indoor conditions during the day in the warm period. An increase in the outside temperature before noon, which culminates in the middle of the day, causes an increase in the indoor temperature with a delayed culmination, depending on the exterior cladding, the orientation of the rooms and the way they are ventilated. As a result, microclimatic conditions in the workplace are worst in the afternoon [55]. This was confirmed by our measurements.

**Figure 5:**
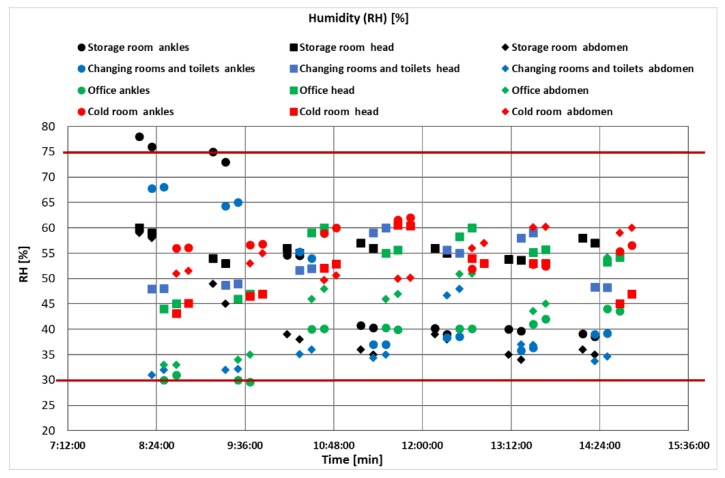
The course of humidity at different height levels in the objectified locations.

The course of changes in humidity measured at individual height levels versus time is shown in Figure 5.

The temperature in dry food storage depends on the nature of the food stored by the manufacturer. In dry warehouses, the allowed relative humidity of the air is within the range of 65–70% [60]. The measured values were mostly at the lower limit of the stated relative humidity. The relative humidity of the air varied depending on the time of measurement and the height of the measuring point. The results show that the majority of cases are less than 60%. The results show an unsuitable space for food storage in terms of constructional solution (the warehouse space was built within 70 years of the last century).

Relative humidity not only affects feelings of comfort but also has a direct impact on human health [61]. Lan et al. [62] presented the experimental measurement of humidity in an office as being around 40% higher than similar values. 

**Figure 6:**
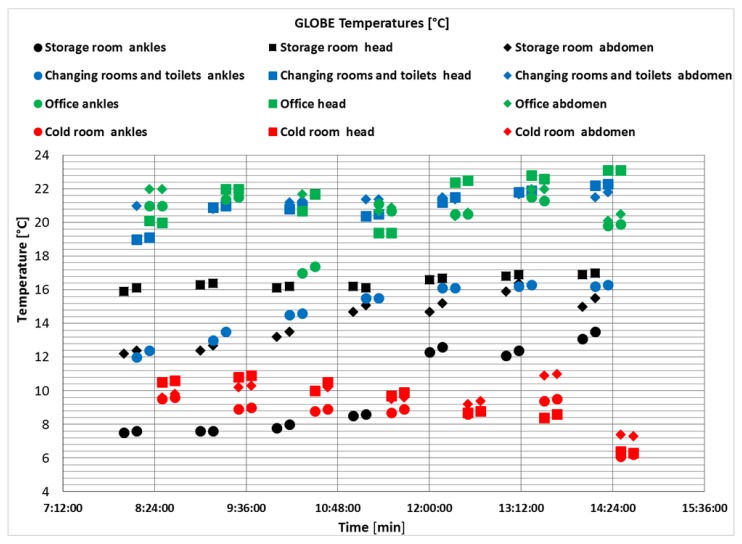
Average temperature values measured by a globe thermometer in the food storage area, in the office, in changing room and in a cold room refrigerator.

The warehouse represented a stable temperature environment from 11:20 am (Figure 6). In the first four hours of measurement, the temperature differences between the ankle and torso heights were more than 4 °C. In the office, the globe thermometer did not capture any significant differences in temperatures at the individual height levels (ankles, torso, head) (Figure 6), which can be considered positive for the employees’ thermal comfort. A change in the air temperature occurred after 12 pm. This change can be attributed to the increase in summer day temperatures and the accumulation of heat in the storage room, which is situated on the south side. Temperature stability has been proven in the changing room (Figure 6), which is also a relaxation room and serves as a heating room in winter. 

## 4. Discussion

The constant values reflect the creation of a comfortable relaxation area. Nevertheless, there was a difference between the ankle and torso (head) temperature levels of almost 4 °C within 12 h, which then decreased to about 2 °C (Figure 5). This was also reflected in the RH indicator in changing rooms (Figure 4).

Constant values at vertical height reflect the creation of a comfortable relaxation area. Nevertheless, the temperature difference between the ankle and the torso (head) was almost 4 °C over 12 h, which was reduced to approximately 2 °C then (Figure 5). This was also reflected in the relative humidity indicator in the changing rooms (Figure 4).

The relative humidity during the measurements reached the highest differences in the storage room at the level of the ankles in the range of 78%–38%, while the temperature increased from the value of 7–12 °C. These results correspond to the nature of the storage area with natural ventilation. Relative humidity evaluated that according limits for employees (30%–70%) exceeded the permissible value at the beginning of the measurement and did not meet the requirements for food stores (65%–70%).

Since the air velocity was ≤ 0.2 m·s^−1^, the operating temperature *t_0_* can be replaced by the resulting value of the globe thermometer *t_g_*. When comparing the results in Table 2, it can be concluded that the temperature values measured by the globe thermometer are higher than the calculated operating temperature in each location. As the temperature rises, this difference increases; in the case of offices, this difference represented up to 8.64%, in the case of the coldest room, which was the cold room refrigerator, the difference was 1.28% higher. In this case, for the specific location in terms of thermal discomfort, it is necessary to consider the calculated values of the operating temperature, which is also recommended by the Slovak methodology defining HTM.

Sakoia and Mochida [63] presented WBGT in their study as an index that approximates the heat accumulation of the human body under standard conditions. If this claim was applied to our measurement results, the assessment of the average effect of heat on the human body during their activity is low (Figure 5). Reached WBGT values during the 6-h measurement interval were low. An employee may perceive thermal discomfort in the warehouse premises, where the measured WBGT_i_ ranged from 4 °C to 14 °C. Although the apparent temperature perceived by employees is low, the parameters of the warehouse were determined by the use of space. The measured parameters cannot be changed, but subjective evaluation can be improved by clothing and work organization.

The warehouse as the main open space had the lowest operating temperature, below the acceptable value of 15 °C, which can be considered as a workplace with thermal discomfort (low apparent temperature perceived by employees) (Table 2). The refrigerator was a critical area, with an operational capacity below 10 °C. 

Necessary increased attention is paid to the evaluation of microclimatic conditions of cultural heritage objects [64,65,66].

The assessed workplaces have different uses and character of work activity. Cases of administrative work and dressing rooms assume an energy expenditure of employees less than 80 W. m^−2^. This type of space is suitable to be equipped with air-conditioning or recuperation systems, with the control of optimal microclimate indicators.

The storage room, where a substantial part of the work is carried out, and the cold room remain problematic. The cold room has specified technical parameters for the purpose of storage.

Therefore, in these spaces, the only optimal solution is the precautions to be taken for proper clothing (more than 1 clo), breaks at work, and rest facilities with hot drinks. Other measures are, for example, more frequent assessment of the health fitness of employees to work in the cold, adaptation process at the start of employment, etc.

### Statistical Evaluation of the Average Measured Values of Microclimatic Conditions in Food Storage

Correct methodological measurement procedure is an important condition for ensuring adequate accuracy. It is not possible to determine an unambiguous and complete procedure to assess systematic errors in the measurement of microclimatic indicators. In standard situations, provided that the prescribed procedure is followed, the measurement uncertainty can be directly determined based on past measurements of the same conditions, but any deviation from the prescribed procedure may call into question the predetermined measurement uncertainty. Nevertheless, completely standard situations are rare, due to a wide variety of measurement conditions, and in most cases determination of the uncertainty associated with the measurement method will apply either to a specific case or to a group of cases measured by the same procedure [52,67].

The measured values of hygrothermal microclimate indicators were statistically processed (Table 2). They were evaluated by descriptive statistics and subsequently, the mean values were calculated according to Formula (2), the mean radiant temperature *t_r,m_* was calculated according to Formula (3) and the operating temperature was according to Formula (4).

The results of the calculation (Table 2) of the mean radiant temperature *t_r,m_* values represent the climatic conditions in the working environment of employees in the food storage environment. The results show that HTM factors (operating temperature) were low.

Offices and changing rooms have achieved optimal conditions for work categorization 2a in the warm period of the year [38]. The warehouse, as the main open space, had a lower operating temperature *t_o_*, below the acceptable value of 15 °C, which can be considered as a workplace with thermal discomfort (low apparent temperature perceived by employees) (Table 2). The cold room refrigerator represented the room with the lowest operating temperature below 10 °C. According to work categorization 2a [38], the energy expenditure of employees is 2.3–2.7 met. The most effective measure is the allocation of personal protective clothing with an insulation of at least 1clo according to CEN Standard EN 15251:2007 [68] and the provision of hot drinks. In the case of prolonged exposure to cold room refrigerator operations, it is necessary to provide heaters for employees and to establish the timetable for the adaptation process to cold microclimatic conditions when new employees are hired.

## 5. Conclusions

Thermal comfort of the employees was evaluated by the operating temperature, including the values of other microclimatic variables. The air velocity in the environment did not exceed 0.20 m·s^−1^. The measurement was carried out in summer, when the ambient temperature was higher than 23 °C. All the indoor temperature parameters were lower. This may be the result of an old warehouse construction (1970s), when the thermo-technical requirements for the microclimate were different than nowadays.

Employees use personal protective equipment that is specified for an administrative employee and a warehouse worker. Despite the summer season, the recommended measures are to use clothing with clothing insulation of at least 1.0 clo in the warehouse. In case of the employees performing work in the cold room refrigerator location, it is necessary to ensure a controlled exposure of employees only for the necessary time periods, while allowing the employees to relax in a heated room and providing them with hot drinks.

The article evaluates the thermal comfort of employees in four different operating processes; in the food storeroom, office, changing rooms and toilettes and cold room. The indicators for evaluation were the operating temperature *t_o_*, including the values of other microclimatic variables. The air velocity in the indoor environment did not exceed 0.20 m·s^−1^. Measurements were made in summer, when the ambient temperature was above 23 °C. All indoor dry temperature parameters were lower. It can be due to the old storage room design (seventies) when the thermo-technical requirements for the microclimate were different than at present.

Employees use personal protective equipment designed for the administrative workers and storage room worker. Despite the summer season, it is recommended to use clothing with an insulation of at least 1.0 clo.

If workers are working in the cold room, it is necessary to ensure the controlled exposure of workers and their presence only for the necessary time periods. Employees can relax in the heated room, with hot drinks provided.

## Figures and Tables

**Table 1:** Characteristics of the premises—overview.

Place of Measurement	Walls	Floor	Celing	Heating	Lighting
Storage room (11 × 17 × 6 m)	Brick	Non-washable flooring	Roofing	Without heaters	Fluor lamps Glass surfaces
Changing rooms and toilets inside the warehouse (3.5 × 9 × 2.6 m)	Brick walls with washable cladding	Washable flooring	Brick ceiling with plaster	Local heat sources	Fluor lamps Glass surfaces
Office in the warehouse (6 × 4 × 2.6 m)	Brick	Washable flooring	Brick ceiling with plaster	Local heat sources	Fluor lamps Glass surfaces
Cold room refrigerator in the warehouse	Brick walls with washable cladding	Washable flooring	Brick ceiling with plaster	Air conditioning unit	Fluor lamps

Freezer room—construction with inside temperature −25 °C.

**Table 2:** Statistical evaluation of the average measured values of microclimatic conditions in the food storage location.

Location	Functions *	Measured Quantities of Hygrothermal Microclimate											*t_g_/t_o_*
		GLOBE *t_g_* (°C)			WBGT_i_ (°C)			WBGT_0_ (°C)			*t_r,m_*	*t_o_*	
		Ankles	Torso	Head	Ankles	Torso	Head	Ankles	Torso	Head	(°C)	(°C)	(%)
Warehouse	Ā	11.9	16.15	17.4	5.7	10.1	13.3	7.4	10.2	13.3			3.16
	R^2^	4.692	0.58	0.156	2.212	0.568	0.236	2.932	0.788	0.188			
	ΔA	2.166	0.761	0.394	1.487	0.754	0.486	1.712	0.888	0.434			
	var	19.691	5.252	2.405	19.830	7.461	3.632	23.139	8.702	3.260			
	Φ	15.15			10.3			10.3			15.85	14.47	
Office	Ā	20.2	21.9	21.3	13.4	15.1	16.2	13.3	14.8	16.54			8.64
	R^2^	2.197	1.856	0.266	1.433	0.445	0.968	2.032	0.642	0.558			
	ΔA	1.482	1.362	0.516	1.197	0.667	0.983	1.415	6.073	0.747			
	var	7.331	6.220	2.418	8.948	4.415	6.073	10.717	5.392	4.517			
	Φ	21.10			15.0			14.8			23.69	19.28	
Changing rooms	Ā	15.4	21.2	21.3	10.6	14.28	15.9	9.6	14.6	15.8			6.68
	R^2^	7.04	0.509	0.086	3.450	0.0656	0.452	4.489	1.202	0.556			
	ΔA	2.635	0.509	0.294	1.857	0.256	0.672	2.119	1.096	0.745			
	var	17.229	3.364	1.376	18.464	1.793	4.228	22.025	7.479	4.719			
	Φ	19.33			13.4			13.2			21.18	18.01	
Cold room refrigerator	Ā	9.1	10.2	10.9	6.6	7.3	8.2	6.6	7.3	8.3			1.28
	R^2^	0.106	0.346	0.922	0.204	0. 285	1.589	0.225	0.265	1.678			
	ΔA	0.326	0.588	0.960	0.451	0.534	1.261	0.475	0.515	1.295			
	var	3.561	5.747	9.740	6.843	7.340	15.226	7.218	7.079	15.533			
	Φ	10.10			7.4			7.4			10.3	9.97	
Work classification derived from [26,27]												15–25	

* Ā—average of daily values, var—coefficient of variation, R^2^—variance, ΔA—standard deviation, Φ—mean value according to Formula (2). GLOBE *t_g_*—globe thermometer temperature, WBGT_i_—wet bulb globe temperature (indoor), WBGT_0_—wet bulb globe temperature (outdoor), *t_g_/t_o_*—the ratio of globe thermometer temperature *t_g_* and operating temperature *t_0_*.
